# The Effects of Perinatal Oxycodone Exposure on Behavioral Outcome in a Rodent Model

**DOI:** 10.3389/fped.2017.00180

**Published:** 2017-08-25

**Authors:** Thitinart Sithisarn, Sandra J. Legan, Philip M. Westgate, Melinda Wilson, Kristen Wellmann, Henrietta S. Bada, Susan Barron

**Affiliations:** ^1^Division of Neonatology, Department of Pediatrics, College of Medicine, University of Kentucky, Lexington, KY, United States; ^2^Department of Physiology, College of Medicine, University of Kentucky, Lexington, KY, United States; ^3^Department of Biostatistics, College of Public Health, University of Kentucky, Lexington, KY, United States; ^4^Department of Psychology, University of Kentucky, Lexington, KY, United States

**Keywords:** prenatal opiate exposure, prenatal oxycodone exposure, behavior, hyperactivity, ultrasonic vocalization, learning and memory

## Abstract

Opiate addiction is now a major public health problem. Perinatal insults and exposure to opiates such as morphine *in utero* are well known to affect development of the hypothalamic–pituitary–adrenal axis of the offspring adversely and are associated with a higher risk of developing neurobehavioral problems. Oxycodone is now one of the most frequently abused pain killers during pregnancy; however, limited data are available regarding whether and how perinatal oxycodone exposure (POE) alters neurobehavioral outcomes of the offspring. We demonstrated that exposure to 0.5 mg/kg/day oxycodone *in utero* was associated with hyperactivity in adult rats in an open field. No significant effects of POE were detected on isolation-induced ultrasonic vocalizations in the early postnatal period or on learning and memory in the water maze in adult offspring. Our findings are consistent with hyperactivity problems identified in children exposed to opiates *in utero*.

## Introduction

The prevalence of opiates and prescription opioid abuse among pregnant women is a major public health concern. Non-prescription opioids are the second most abused illicit substance and one of the most commonly abused opiate pain relievers is oxycodone (1). In 2015, an estimated 10.4% of the population over 12 years of age used oxycodone products. Importantly, substance use in pregnant women and subsequent fetal exposure to drugs have been linked to adverse health effects for the maternal–fetal dyad. Opiates can affect the developing fetus directly or indirectly through various mechanisms. Opiates can cross the placenta (2–4) and act directly on fetal opioid receptors. Opiates can also enhance maternal secretion of cortisol in the mother or stimulate the secretion of stress hormones in the fetus (5), which can pose long-term effects to the developing fetus (6) and the hypothalamic–pituitary–adrenal (HPA) axis of the offspring (7, 8). In addition, because the HPA axis has important roles in programming neurobehavioral development (9) and dysregulation of the HPA axis has been linked to several neuropsychiatric disorders, such as anxiety (10), depression (11, 12), ADHD (13), and learning/memory deficits (14, 15, 16), any opioid-induced changes in the HPA-axis may also be associated with short- and long-term behavioral problems.

Both human and animal studies have shown that exposure to opiates has deleterious effects on neurodevelopmental outcomes. However, most of them used other opiates, including morphine and heroin, which are mu-opioid receptor agonists, rather than oxycodone, a semisynthetic putative kappa opioid receptor (KOR) and partial mu opioid receptor agonist. Because illegal oxycodone use during pregnancy continue to rise, and the effects of opiates on the HPA axis differ depending on the type of opiate (17, 18, 19), it is important to determine the effects of perinatal oxycodone exposure (POE) on the neurobehavioral outcome of the offspring.

The current study was designed to examine the potential effects of POE on three diverse behaviors; isolation induced ultrasonic vocalizations (USVs) in neonatal pups followed by activity levels in adolescent rats and spatial learning in young adult rats. Isolation-induced USVs are the responses of young rat pups when separated from their mothers (20, 21). USVs are considered an adaptive response of the pup and these USVs correlate directly with distress and/or anxiety in the rat pup (22). USVs elicit maternal behavior and play an important role in the interaction between the pup and the dam (21). USV cues may be comparable to the crying sounds of human infants (23), which have been used to identify infants at risk for poor neurobehavioral outcomes (23, 24). Although existing data on the effects of prenatal opiate exposure on isolation-induced USVs remain limited, neonatal exposure to alcohol (25) or other illicit drugs, such as cocaine, can alter many USV characteristics (26). Therefore, we hypothesized that rat pups prenatally exposed to oxycodone would have an increased latency to the first USV and decreased USV numbers per minute.

The second testing paradigm used was an open field test, which has been widely used to study hyperactivity, anxiety, and stress in animals (27, 28, 29, 30). Although prenatal exposure to morphine was not associated with increased locomotor activity in the open field in rats (31, 32), human studies have shown that children exposed to opiates *in utero* manifest hyperactivity, impulsivity, and attention problems, whether the mothers were in opiate maintenance therapy or were polysubstance users (33, 34). Therefore, we hypothesized that POE would be associated with hyperactivity in the open field in rat offspring.

Learning and memory were assessed in a water maze. Children prenatally exposed to opiates have learning problems and lower scores on neurodevelopmental tests (35, 36, 37). In rats, prenatal exposure to morphine is associated with learning and memory deficits (38, 39). Thus, we hypothesized that POE would impair learning and memory of the offspring. To the best of our knowledge, these studies are the first to look at the effects of POE on these behavioral measures.

## Materials and Methods

### Experimental Design: Animals and Prenatal Treatments

The study protocol was approved by the University of Kentucky Institutional Animal Care and Use Committee. Virgin female Sprague Dawley rats (Harlan, Indianapolis, IN, USA) weighing 194–223 g were individually housed at 22–25°C and maintained in a 14L:10D photoperiod (lights on at 0500 h) room with regulated 30–70% humidity. Rat chow and water were provided *ad libitum*.

Once released from quarantine, the females were fitted with a right atrial cannula connected to a subcutaneous (S.C.) access port implanted between the shoulder blades. The rats were allowed to recover for 1 week. To determine estrous cycles, vaginal lavages were obtained daily. Each female was group housed with a proven breeder male for breeding 1 week after cannulation. Gestational day (GD) 0 was designated as the day that sperms were detected in the vaginal smear, and the females were individually housed thereafter. Foster dams were bred at the same time and remained untreated throughout their gestation.

The cannulae were flushed daily *via* the S.C. port with sterile heparinized saline (0.4 cc, 100 IU/ml) until GD 8. From GD 8–21, the experimental dams were divided into three treatment groups that received either oxycodone (Mallinckrodt, St. Louis, MO, USA) at a low (OXY-L, 0.5 mg/kg/day, *n* = 5) or high dose (OXY-H, 2.0 mg/kg/day, *n* = 12) or an equivalent volume of vehicle [control (CON), normal saline solution (NSS, 1.0 ml/kg/day), *n* = 12] from GD 8–21. These solutions were slowly injected i.v. over 10 min *via* the S.C. access port manually.

On postnatal day (PD) 1, the pups were counted and weighed. Oxycodone or NSS was also administered on PD 1, 3, and 5 to the dams because brain development during early postnatal life overlaps the human “third trimester” brain growth spurt and to prevent maternal withdrawal symptoms that might affect maternal nursing behavior. On PD 2 all litters were adjusted to contain 10–11 pups with equal numbers of male and female pups when possible. At 1700 h on PD 5, all pups in each litter were transferred to an untreated foster dam to minimize exposure to altered maternal rearing behavior that has been described in rat dams after exposure to opiates (40). To preclude potential litter effects, only one male and one female from each litter were included in the behavioral studies (41). The pups were weighed daily and weaned at PD 25, when they were separated by sex.

### Experimental Design: Behavioral Tests

#### Ultrasonic Vocalizations

Ultrasonic Vocalizations were determined according to published procedures (42). In brief, an ultrasonic bat detector (Ultra Sound Advice Model #S-25, UK—http://www.ultrasoundadvice.co.uk) set at 40 kHz with a condenser microphone (SM-1) set 21.5 cm above the test cage floor was used. The output was recorded on a SONY #WM-D8C Cassette Recorder using low noise cassette tapes. On PD 14, the pups in each litter were separated from the dams, remaining in their home cage and were brought to a neighboring test room one litter at a time. Their cages were placed halfway on a heating pad to provide warmth. During testing, the dam was placed in a new cage in the same test room. CON offspring (*n* = 6) and OXY-L (*n* = 4) and OXY-H (*n* = 8) rats underwent USV testing. The number of pups in the OXY-L group was smaller due to smaller numbers of dams in this group and limited numbers of pups available for all experiments that were performed. During USV testing, pups were isolated one at a time in a clean cage for 6 min during which USVs from the pup were recorded. A fan was used to provide white noise during testing. Upon completion of USV testing for the entire litter, the dam was returned to the home cage and the home cage was returned to their original animal room.

Assessment of USV: audio data for each 6-min test period were individually scored for latency to vocalization (seconds) and number of USVs per minute per test period. Scoring was performed independently by two experimenters who were blind to the treatment groups and used a stopwatch and clicker counter. Similarity of their scores was compared and indicated that the reliability between experimenters was greater than 90%.

#### Open Field Test

On PD 43, a group of offspring that had not undergone USV testing were transferred to another building, where they were housed for at least 7 days prior to subsequent behavioral testing. This was an independent group of subjects, i.e., not the same offspring that had previous USV testing. The open field test was preceded by 5 days of habituation to the new surroundings followed by 2 days of 3-min handling periods and weighing. CON offspring (*n* = 30, 19 males, 11 females) and OXY-L (*n* = 8, 4 males, 4 females) and OXY-H (*n* = 32, 17 males, 15 females) rats underwent open field tests on PD 50–51. The number of pups in the OXY-L group was smaller due to limited numbers available after other experiments (not described herein) were performed. The open field apparatus was a circular chamber 36 cm height and 58 cm diameter. This circular chamber was used to prevent thigmotaxis or excessive time in corners (43). In addition, the open field was divided into two zones, with the center zone representing 25% of the total area. All testing was conducted in a test room with white noise to reduce external distractions. On days 1 and 2 of testing, two rats were transferred in separate cages and brought into the test room for a 10 min habituation period. Each animal was then placed in a separate open field, and their activities were recorded with a Polytracker^®^ (San Diego Instruments, San Diego, CA, USA) for 30 min. The animals were then returned to their home cages in the colony room upon completion of testing.

Activity was recorded in 5 min blocks across the 30 min test period for each day. Total distance traveled (cm), distance traveled in the center zone, and the ratio of distance traveled in the center zone to total distance traveled or to distance in the outer zone were determined.

#### Water Maze

On PD 55–56, two groups of offspring, CON (*n* = 20, 11 males, 9 females) and OXY-H (*n* = 20, 10 males, 10 females) were tested in the water maze. Rats from the OXY-L group were not tested due to the limited numbers of pups. This experiment was modified from a procedure previously described (43). The apparatus was a 130 cm × 90 cm × 40 cm black Plexiglas chamber, divided such that several divergent paths, each 18 cm wide, branched off from the central start area. The apparatus and methodology were modified from von Euler et al. (44). Water temperature was maintained at 76° ± 2°. In this test, rats must learn to swim and make three successive right/left choices to a platform that is submerged below the water level and invisible; the water was made black with the addition of non-toxic black tempera paint to obscure the submerged platform. A plastic sheet surrounded the maze, reducing extra-maze cues, including the experimenter. A major advantage of this maze is that control animals can learn the maze in a single day. Movement in the maze was recorded using a video tracking system (SMART program; Panlab, S.L.) (43).

On the first trial, the rat was placed in the maze and allowed to swim freely. If the animal did not reach the platform after 1 min, it was guided to the platform. After 5 s on the platform, the animal was transferred to a cage warmed by a heat lamp (25 W) for 30 s. Trials were repeated until the animal completed two consecutive trials without errors (wrong turns) or a total of three successful trials. The number of trials required to reach either criterion was recorded as the outcome measure. The next day the rats were tested identically for 24 h to determine retention.

### Statistical Analyses

Statistical analyses were considered significant if *p* < 0.05. Multilevel linear regression models or linear mixed effects models were used to analyze USVs, open field and water maze data. Such models accounted for potential litter effects and correlation among outcomes from the same pup over time. The models were fitted using restricted maximum likelihood to test for the impact of treatment group, gender, time, and any potential interactions these variables have on the mean values for the data. Due to skewness and outliers of the data, a natural log transformation was applied when appropriate. The Kenward and Roger (45) approximation was used to estimate standard error and degrees of freedom. Variables (trial days, sex, and treatment groups) were treated as categorical and backward elimination at the 5% significance level was utilized. Kruskal–Wallis tests were applied to compare litter sizes and number of males per litter between treatment groups. A linear mixed-effects model was applied to compare differences in body weight between groups with treatment group, gender, PD, and the interaction of gender and PD included as predictors of weight. Tests were two-sided and were conducted in SAS version 9.4 (SAS Institute, Cary, NC, USA).

## Results

### Litter Size and Body Weight

Perinatal oxycodone exposure did not affect litter size or number of males and females per litter (Table 1).

**Table 1:** Litter size, the number of male and female rat pups per litter, maternal weight gain during pregnancy and birth weight of the pups and weight of the young adult offspring at the neurobehavioral tests (g ± SEM).

Variable (number of litter)	CON (*N* = 11)	OXY-H (*N* = 13)	OXY-L (*N* = 5)	*p*-Value
Total number of pups per litter	13.3 (14) ± 2.9	12.3 (13) ± 4.4	14.4 (14) ± 1.5	n.s.
Number of females per litter	5.9 (6) ± 2.1	6.5 (7) ± 3.1	6.0 (6) ± 1.0	n.s.
Number of males per litter	7.4 (8) ± 3.0	5.8 (6) ± 2.5	8.4 (8) ± 0.5	n.s.
Maternal weight gain during pregnancy, mean ± SEM (g)	153 ± 7.3*	113.4 ± 10.0*^#^	156.1 ± 9.0^#^	*p = 0.01, CON vs OXY-H, diff 39.7 g ^#^*p* = 0.023, OXY-H vs OXY-L, diff 42.8 g
Birth weight, mean ± SEM (g)				
Male (M)	M:6.4 ± 0.2	M: 6.2 ± 0.3	M: 6.1 ± 0.4	n.s.
Female (F)	F: 6.0 ± 0.2	F: 5.8 ± 0.3	F: 5.6 ± 0.5	n.s.
Body weight during neurobehavioral tests (adult)	M: 223 ± 6.7	M: 250 ± 1.5	M: 210 ± 8.0	n.s.
	F: 169 ± 5.4	F: 177 ± 3.5	F: 175 ± 4.9	

*CON, control; OXY-H, oxycodone high dose; OXY-L, oxycodone low dose*.

**Figure 1:**
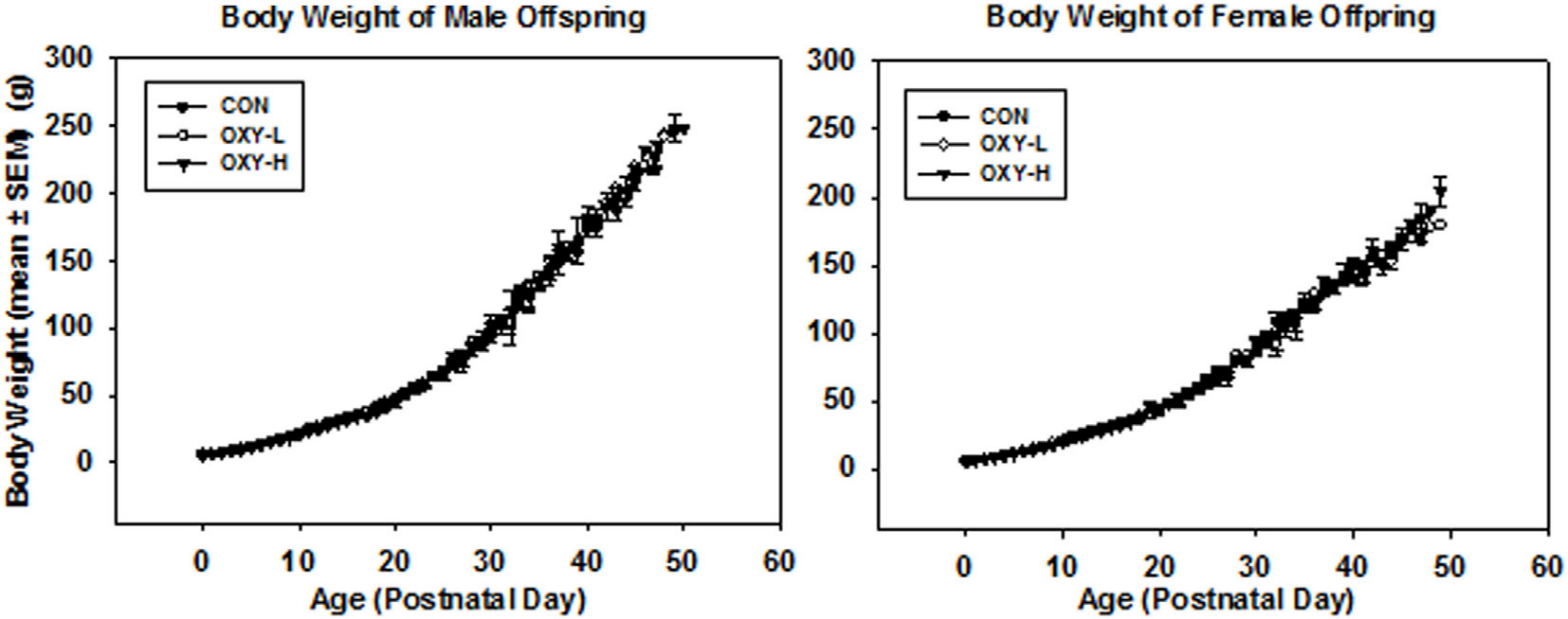
Body weight of male and female offspring: left panel: body weight of male pups from birth through postnatal day (PD) 50 in control (CON, closed circles, *n* = 81), oxycodone low dose (OXY-L, open circles, *n* = 42) and oxycodone high dose (OXY-H, closed triangles, *n* = 76) groups. Right panel: body weight of female pups from birth through PD 50 in control (CON, closed circles, *n* = 65), OXY-L (open circles, *n* = 30) and OXY-H (closed triangles, *n* = 84) groups.

Although the average maternal weight gain during pregnancy was different between groups (*p* = 0.005), perinatal oxycodone treatment did not affect mean birth weight of male or female pups weighed within 24 h after birth (Table 1). Dams in the OXY-H group gained less weight than CON dams (*p* = 0.01) and OXY-L dams (*p* = 0.023) (Table 1). Oxycodone treatment did not affect weight gain of the pups; body weights up to PD 50 were not different between groups (*p* = 0.61). However, there was an interaction between gender and postnatal age (*p* < 0.0001); males and females had different growth trajectories over time, as expected (Figure 1).

Body weights of both male and females were similar across treatment groups in the rats tested in the open field and water maze (*p* = 0.43), the females weighing less than the males as expected (*p* < 0.0001) (Table 1). The body weight of the pups at the time of USV testing (PD14) (g ± SEM) were 30.9 ± 1.8 in male and 29.1 ± 1.6 in female CON group; 30.2 ± 1.9 in male and 28.1 ± 1.9 in female OXY-L group; and 30.1 ± 1.4 in male and 28.7 ± 1.1 in female OXY-H group.

### Ultrasonic Vocalizations

**Figure 2:**
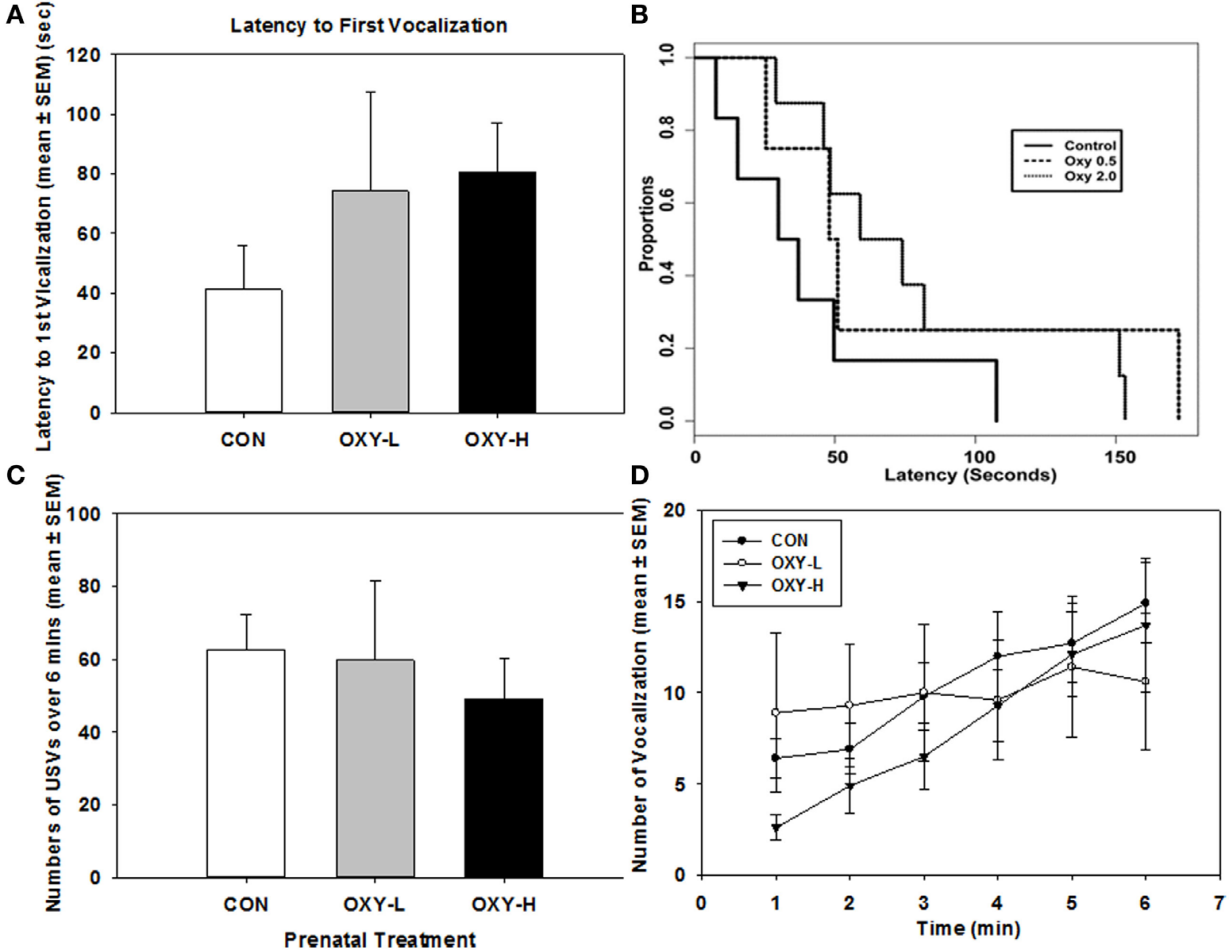
**(A)** Latency to the first vocalization of rat pups; **(B)** Kaplan–Meier plot of latency to the first ultrasonic vocalizations (USVs) in rat pups in control (CON, solid line), oxycodone low dose (OXY-L, long dashed line), and oxycodone high dose (OXY-H, dotted line) groups; **(C)** total number of vocalizations of rat pups during USV test in CON (white bar; *n* = 6), OXY-L (gray bar; *n* = 4), and OXY-H (black bar; *n* = 8) groups; and **(D)** number of USVs per minute across the testing period in CON (closed circles), OXY-L (open circles), and OXY-H (closed triangles) groups.

There were no significant sex differences so results were collapsed across sex for subsequent USV analyses. There was no significant difference in the latency to the first vocalization between treatment groups; however, there was a trend for longer latencies in OXY-exposed rats, Figure 2A. Latency to the first USV as plotted in Kaplan–Meier Plots is shown in Figure 2B, a point on the plot denotes the estimated probability of having a first USV after the given time point. Although there was a trend for prenatal oxycodone-exposed pups to display longer latencies to their first USV after isolation, there were no significant differences between treatment groups (*p* = 0.25), likely due to small sample sizes, Figure 2B.

Total USVs across time also did not differ among the three treatment groups (*p* = 0.85), Figure 2C. The number of USVs increased across time in all treatment groups (*p* = 0.0004); however, there was no significant effect of perinatal OXY exposure (*p* = 0.32), Figure 2D.

### Open Field Test

#### Analysis of Total Distance Traveled in 30 Min

**Figure 3:**
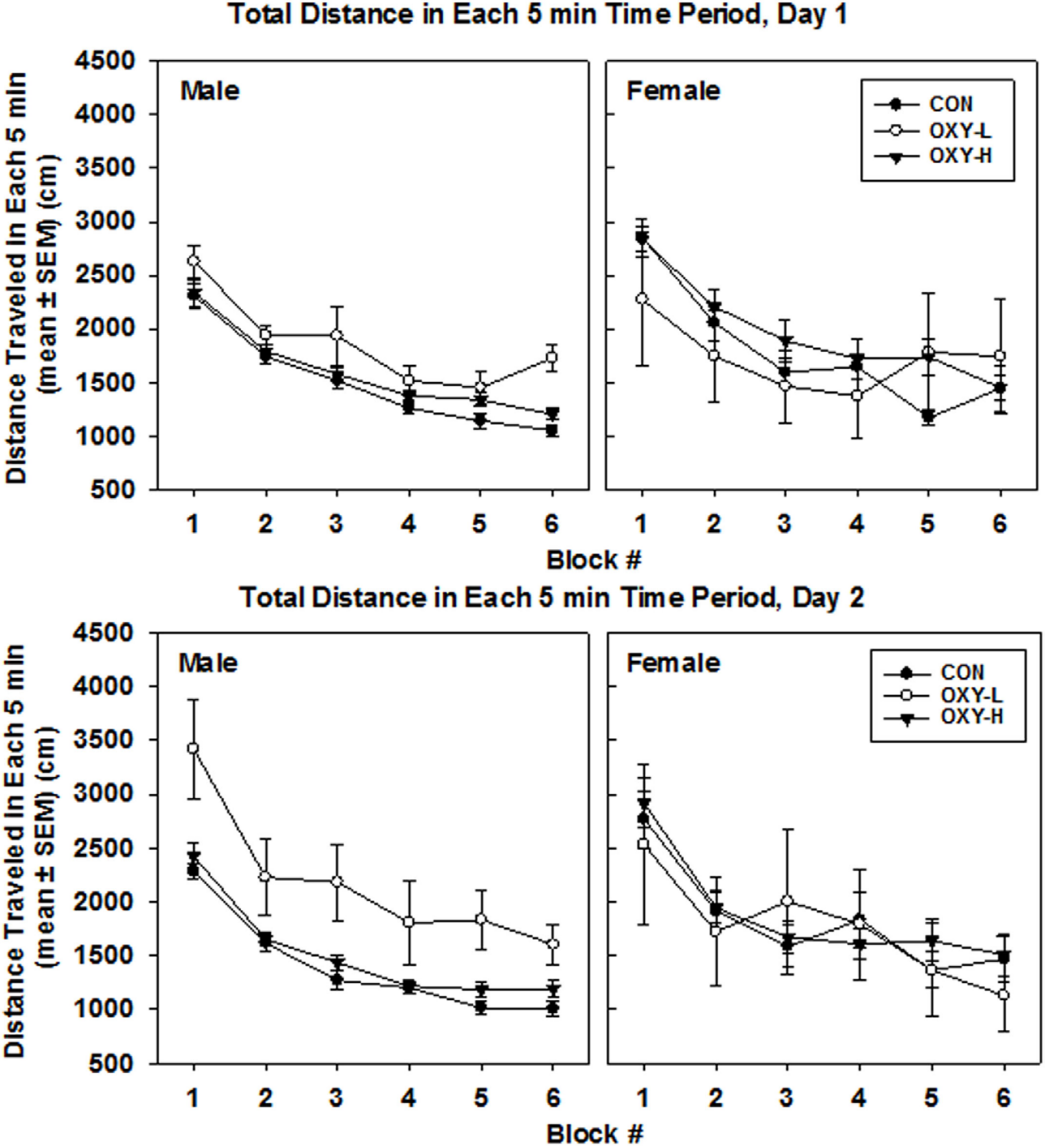
Total distance traveled in each 5 min time block in the open field on test day 1 (upper panel) and test day 2 (lower panel), by males (left panel) and females (right panel) in control (CON, closed circles; male *n* = 19, female *n* = 11), oxycodone low dose (OXY-L, open circles; male *n* = 4, female *n* = 4), and oxycodone high dose (OXY-H, closed triangles; male *n* = 17, female *n* = 15) groups.

Analysis of the raw data indicated that there were no statistically significant differences in total distance traveled among rats in CON, OXY-L, or OXY-H groups (*p* = 0.26). There was also no effect of test day on the total distance traveled (*p* = 0.48). No interactions were significant. Female rats traveled a greater total distance compared to males [traveled 1,654 cm further than males, 95% CI: (539, 2,768), *p* = 0.004]. After a natural log transformation of the data, because of outliers and skewness, it was similarly determined that females traveled a greater total distance than males (*p* < 0.0001). In addition, this transformation of the data revealed that the OXY-L rats traveled farther than rats in both other treatment groups (vs OXY-H, *p* = 0.011; vs CON, *p* = 0.0004, Figure 3). This difference was likely largely driven by OXY-L-treated males on day 2.

There was no main effect of perinatal treatment or test day (*p* = 0.10 and *p* = 0.07, respectively) on the total distance per 5 min block. However, females travel 241 cm further than males in any given 5 min period (*p* = 0.003). The mean total per 5 min time block decreased over time (*p* < 0.0001), Figure 3.

#### Analysis of Total Distance Traveled in the Inner Zone, Outer Zone, and Mean Ratio of Distance Traveled in the Inner/Outer Zone

##### The Inner Zone

**Figure 4:**
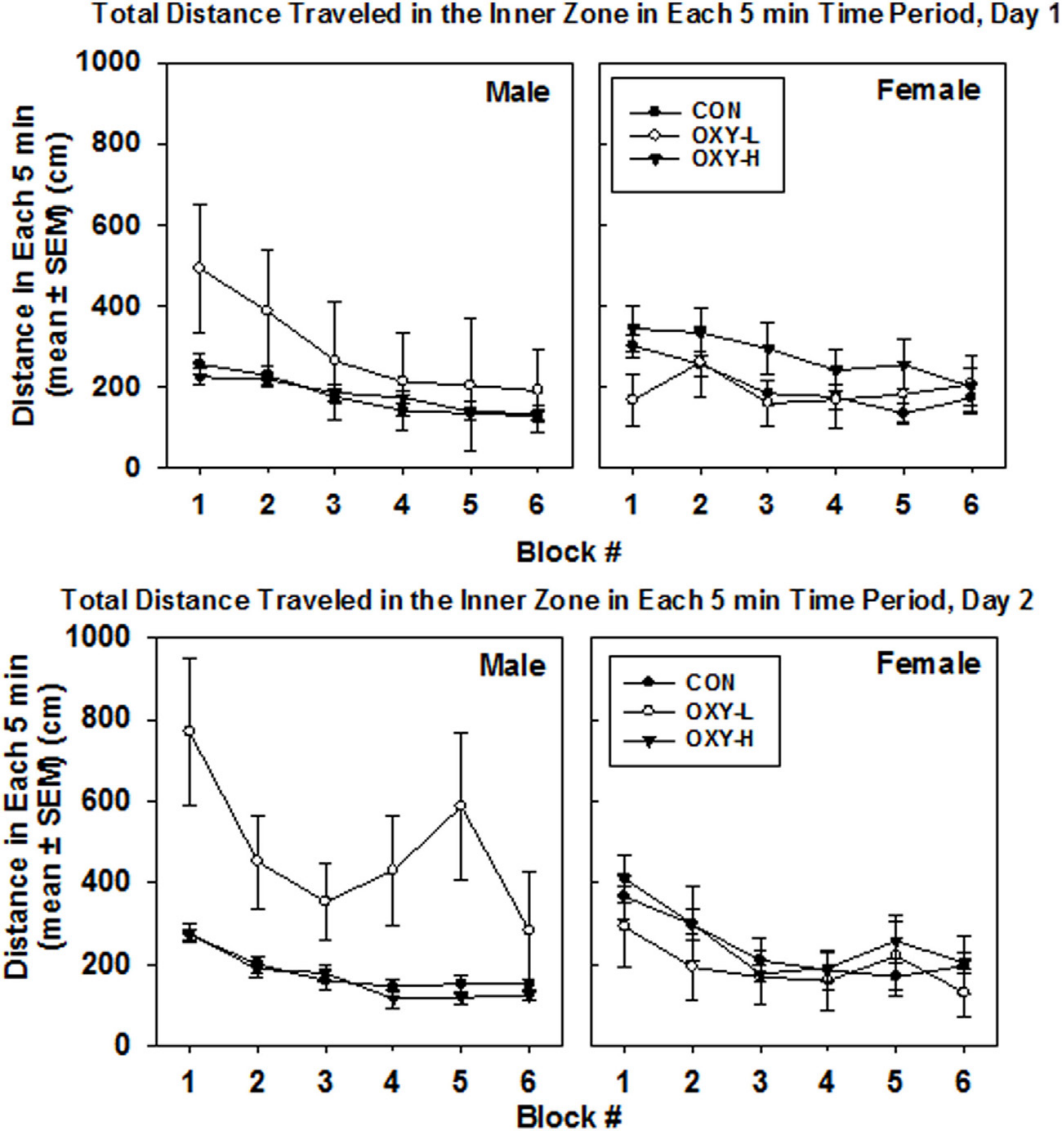
Total distance traveled in inner zone in each 5 min time block in the open field on test day 1 (upper panel) and test day 2 (lower panel), in males (left panel) and females (right panel) in control (CON, closed circles), oxycodone low dose (OXY-L, open circles), and oxycodone high dose (OXY-H, closed triangles) groups.

Rats exposed to OXY-L traveled more in the inner zone than rats in the other two treatment groups (OXY-L vs OXY-H, *p* = 0.011; vs CON, *p* = 0.003), likely due to the travel of the OXY-L-treated males on day 2, Figure 4. The distance traveled in the inner zone did not differ between days (*p* = 0.61), and no interactions were significant.

Oxycodone treatment did not affect the distance traveled in the inner zone per 5 min block, Figure 4. The large standard errors were likely due to the small sample size.

##### Outer Zone

**Figure 5:**
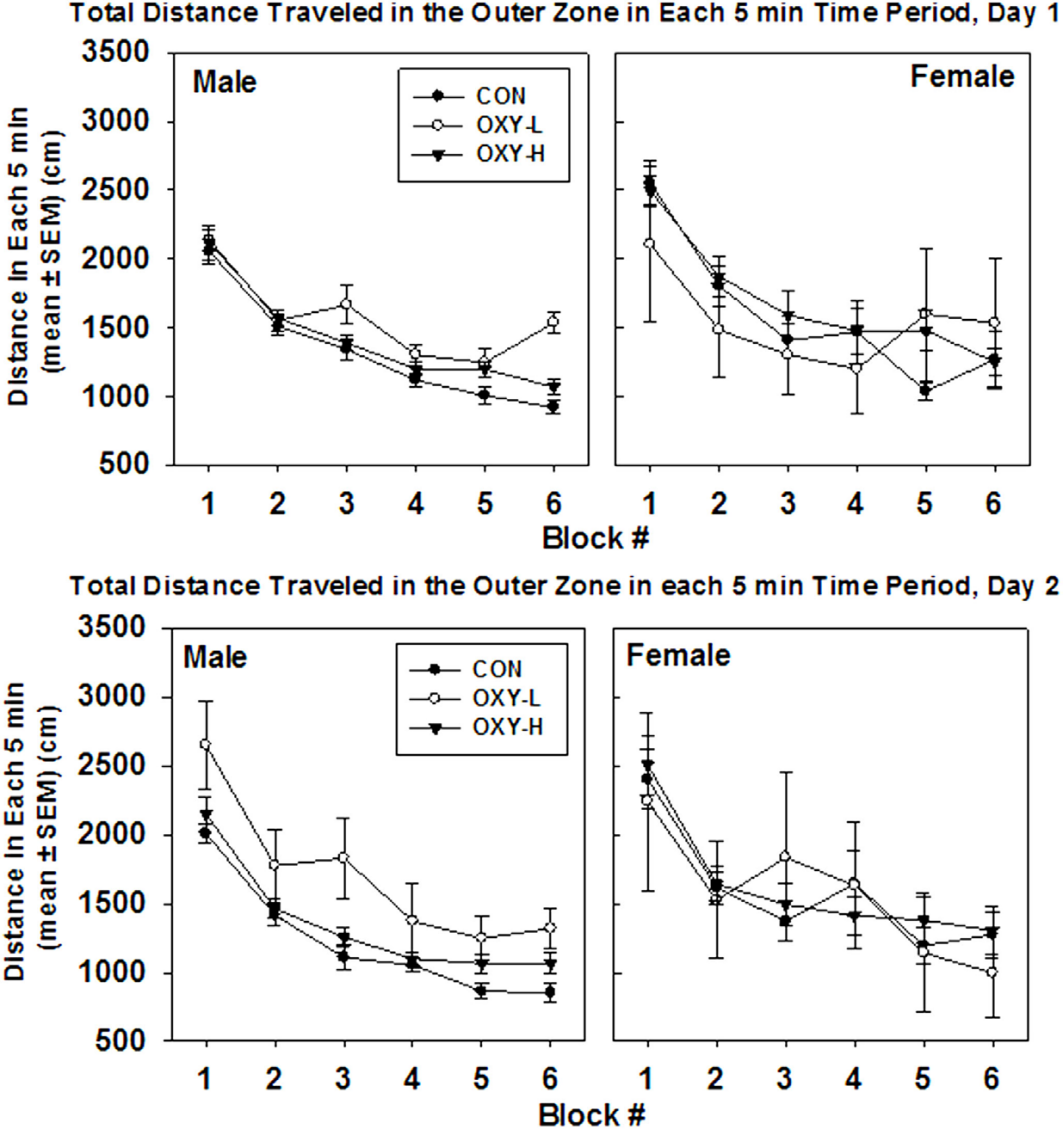
Total distance traveled in outer zone in each 5 min time block in the open field on test day 1 (upper panel) and test day 2 (lower panel), by male (left panel) and female (right panel) rats in control (CON, closed circles), oxycodone low dose (OXY-L, open circles), and oxycodone high dose (OXY-H, closed triangles) groups.

There were no significant interactions. Neither oxycodone treatment nor testing day impacted the mean total outer zone distance (*p* = 0.35 and *p* = 0.29, respectively), Figure 5. After natural log transformation and removal of two outliers, there were main effects of gender (*p* < 0.0001) and treatment group (*p* = 0.005). Namely, males traveled to the outer zone less than females (*p* = 0.003) and OXY-L rats traveled more in the outer zone than the CON group (*p* = 0.002, Figure 5). There was no difference in outer zone distance traveled between either CON or OXY-L compared to OXY-H, Figure 5.

There were no interactions in outer zone distance per 5 min and no effect of oxycodone treatment (*p* = 0.15). Females travel 194 cm further than males in the outer zone in any given 5 min period (*p* = 0.01), Figure 5. In addition, rats tended to travel 113 cm further on the first test day in any given 5 min time period (*p* = 0.032), Figure 5. The mean total distance traveled in the outer zone per 5 min period decreased over time as expected (*p* < 0.0001), Figure 5.

#### The Ratio of the Distance Traveled in the Inner Zone to Outer Zone

**Figure 6:**
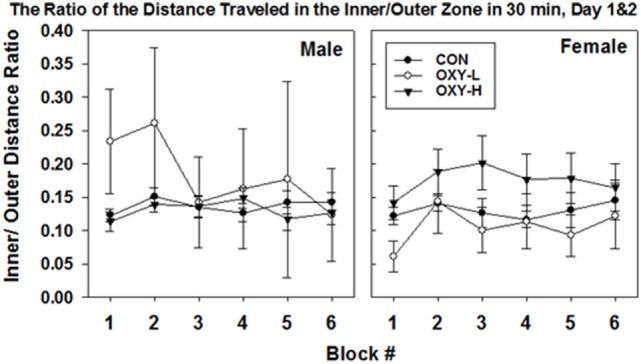
The ratio of the distance traveled in the inner/outer zone in 30 min in the open field (mean ± SEM) by male (left panel) and female (right panel) rats in control (CON, closed circles), oxycodone low dose (OXY-L, open circles), and oxycodone high dose (OXY-H, closed triangles) groups. Male OXY-L rats had overall higher ratios of distance traveled in the inner to outer zone for each 5 min time block across time compared to other treatment groups (OXY-L vs CON: *p* = 0.04; OXY-L vs OXY-H = 0.007).

The ratio of activity in the center zone vs outer zone of the field is a potential marker of motor impulsivity and/or anxiety (46). There were no differences in mean ratios across test days (*p* = 0.18) but there was a treatment group × sex interaction (*p* = 0.003; sex × treatment group interaction). Specifically, the estimated mean ratio of the distance traveled in the inner to outer zone of male OXY-L rats which was 0.245, was 0.095 larger than that of the CON, *p* = 0.008, and 0.112 larger than for OXY-H, *p* = 0.002 (Table 2). This pattern differed across sex; no differences in the inner:outer ratio were observed in the females (estimated mean ± SEM for days 1 and 2 in CON: 0.13 ± 0.01, OXY-L: 0.10 ± 0.05, and OXY-H: 0.17 ± 0.02) (Table 2). The differences in the males persisted when the ratios of distance traveled in the inner to outer zone for each 5 min time block were analyzed (OXY-L vs CON: *p* = 0.04; OXY-L vs OXY-H = 0.007), Figure 6.

**Table 2:** The mean ratios of the distance traveled in the inner to outer zone in male and female rats in 30 min testing period.

Test day	CON (SEM)	OXY-L (SEM)	OXY-H (SEM)
**Male**			
1	0.13 (0.01)	0.19 (0.09)	0.13 (0.01)
2	0.15 (0.01)	0.27 (0.07)	0.13 (0.01)
Estimated mean day 1 and 2	0.14 (0.01)	0.24 (0.07)*	0.12 (0.01)
**Female**			
1	0.13 (0.01)	0.10 (0.04)	0.17 (0.03)
2	0.14 (0.02)	0.11 (0.04)	0.17 (0.02)
Estimated mean day 1 and 2	0.13 (0.01)	0.10 (0.05)	0.17 (0.02)

*p < 0.05 {the estimated mean ratio of distance traveled in the inner to outer zone of male OXY-L rats which was 0.245, was estimated to be 0.095 larger than that of the CON [95% CI: (0.027, 0.162), *p* = 0.008], and 0.112 larger than for OXY-H [95% CI: (0.042, 0.1830, *p* = 0.002)]}.

*CON, control; OXY-H, oxycodone high dose; OXY-L, oxycodone low dose*.

### Water Maze

**Figure 7:**
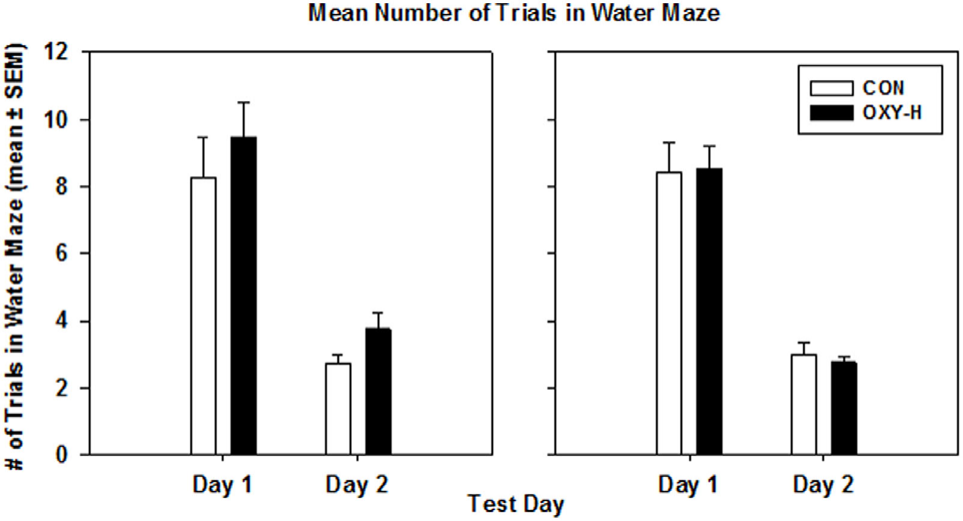
The mean number of trials in the water maze in male (left panel) and female (right panel) rats in control (CON, white bars, *n* = 20, 11 males, 9 females) and oxycodone high dose (OXY-H, black bars, *n* = 20, 10 males, 10 females) group.

Using a multivariate repeated measures Gaussian linear model to analyze the data with trial days, treatment groups and sex as predictor categories, there was no effect of oxycodone or sex on the mean values of the average number of trials until criterion performance was reached on both days (*p* = 0.62). The expected decrease in the mean values of the average number of trials on day 2 was observed in all groups (*p* < 0.0001) (Figure 7).

## Discussion

We explored the effects of POE on behavioral outcomes using three behavioral tests (USV, open field, and water maze) in juvenile, adolescent, and young adult rats. POE increased locomotion and preference for the center in the open field, consistent with hyperactivity. However, we did not find any effects of POE on USVs of rat pups when separated from their mothers or on learning and memory in the water maze.

### Open Field Test

Oxycodone low dose rats were hyperactive and traveled more in the inner zone of the open field compared to CON and OXY-H rats, resulting in a higher mean ratio of inner:outer zone distance traveled by the OXY-L males. Thus, perinatal exposure to oxycodone was associated with hyperactivity behavior in adolescence. In addition, the increase in the ratio of inner zone:total distance traveled suggests that perinatal oxycodone reduced anxiety-like behavior and decreased normal species-typical thigmotaxic behavior in a new environment. Alternatively, these differences might reflect overall hyperactivity with a failure to inhibit entries to the center. It remains to be determined whether the increased travel in the center is due to deficits in hyperactivity, impulse control, or reduction in anxiety.

Our finding that perinatal exposure to oxycodone was associated with hyperactivity in the offspring is in agreement with human studies that identify hyperactivity, impulsivity, and attention problems in children exposed to opiates *in utero* (33, 34). In animal models, however, the results are more conflicting due to differences in studied drugs, which were mainly morphine, as well as in paradigms, ages, instruments, and end points. For example, in one study, postnatal handling but not prenatal morphine exposure increased locomotor activities in the open field in adult male and female rats (31). In contrast, others reported that postnatal stress but not prenatal exposure to morphine increased locomotor activity in the open field (32). Thus, morphine yields different results than oxycodone in the open field. However, it should be noted that the effects of morphine are contradictory among studies and differ among tests. Thus, prenatal exposure to morphine was associated with increased anxiety-like behavior in an elevated plus maze (EPM) and reduction in time spent in the lit side of a light/dark box (L/D box) (47). Others reported that rats prenatally exposed to morphine exhibited decreased anxiety-like behavior in the EPM and L/D box with no differences in distance traveled over 30 min in the open field (48). Another study reported no significant anxiogenic effect of prenatal morphine exposure determined in the EPM (49). Finally, prenatal exposure to several opiates, such as methadone, buprenorphine, and most effectively morphine, increased anxiety-like behaviors in the light–dark transition test, with no effect on locomotor activity in an open field (50).

A possible mechanism by which prenatal exposure to opiates could result in hyperactivity may involve changes in multiple neurotransmitter signaling pathways such as dopaminergic pathways and the HPA system. It is well described that opioids have a significant role in controlling the release of dopamine and acetylcholine in the key reward regions of the brain including the ventral tegmental area (VTA) and the nucleus accumbens (51, 52, 53, 54). Long-term exposure to opiates leads to both structural and biochemical changes in the mesolimbic dopaminergic system; for example, a reduction in the cell size of dopaminergic neurons in the VTA (55), and increased levels of tyrosine hydroxylase, which is the rate-limiting enzyme in the synthesis of dopamine in the VTA (56). In addition, convincing evidence suggests that the impairment of dopamine-mediated development and the monitoring of motivated behavior and reward-related memory formation might be associated with ADHD symptoms (57, 58). Thus, taken together, it is possible that perinatal exposure to opiates may disrupt the normal development of dopaminergic reward-related circuits leading to hyperactive behavior. However, this speculation remains to be further elucidated.

Changes in the HPA system have also been linked to ADHD symptoms. In children, both reduced basal cortisol secretion and cortisol hyporeactivity have been associated with hyperactivity/impulsivity or a combined type ADHD (59). An abnormal diurnal rhythm and less effective negative feedback mechanisms after a dexamethasone suppression test were also identified more frequently in the children with ADHD that were severely hyperactive compared to those with milder symptoms (60). In agreement with many community-based studies, in which ADHD is more prevalent in males (61), in our study hyperactivity was more notable in the male OXY-L group. Interestingly, in adults with ADHD, although the basal salivary cortisol levels were not different, cortisol levels 20-min after a mental cognitive stress test were higher than those in healthy adult controls (62). These findings are similar to our observations that POE is associated with increased CRH-induced ACTH release (8) and with increased corticosterone reactivity to restraint stress in adult female rats (unpublished). However, we did not measure corticosterone, the major circulating glucocorticoid in rats, during the behavioral tests in the current studies. The discrepancy between hyperactivity in males and increased levels of corticosteroids in females could be due to the methods used to produce the stress in each study, relatively small sample sizes, and/or the gender of the participants. Referral biases and methodological difficulties were present in other human studies (63, 64). Thus, the relationship between POE, hyperactive behaviors, and abnormal HPA axis function that may interact with gender warrants further investigations, which should include the measurement of corticosterone during and after neurobehavioral testing. The abnormal HPA axis response to stress may be a promising candidate for use as a biomarker for early detection and treatment of ADHD in infants prenatally exposed to oxycodone/opiates.

Data from both animal and human studies suggest that early nutritional stress and malnourishment are associated with anxiety, high impulsivity, and attention problems (65). Although the weight gain of the OXY-H dams was significantly lower than that in the other groups, the weight gain of the OXY-L dams was actually comparable to that in the CON group. In addition, birth weights of the pups were comparable in all groups. Therefore, the hyperactivity in the open field of the OXY-L group could not solely be explained by poor maternal nutritional status.

### Ultrasonic Vocalizations

Although there was a trend toward increased latency to first vocalization, oxycodone treatment had no effect on this end point or on the number of vocalizations per minute. The lack of differences in these USVs parameters may be due in part to the relatively low sample size in the OXY-L group. To our knowledge, there are no reports thus far on the effects of perinatal oxycodone on USVs In contrast to our results, however, perinatal cocaine treatment decreased the number of USVs on PD 1 and on PD 21, but this effect was not observed on PD 14 (66). Therefore, it is possible that the lack of an effect of oxycodone in our study occurred because testing was limited to PD 14. In fact, there are other types of USVs based on frequency and duration that are elicited as the rat becomes more mature (67). These include a 22 kHz USV emitted by juvenile and adult rats in response to predators and pain indicating a negative affective state (68, 69), and a 50 kHz USV emitted in adult rats in response to rewarding stimuli, expressing a positive affective state (70, 71). So it is also possible that POE may affect these other types of USVs that were not tested at an older age.

### Water Maze

We did not find any effects of POE on spatial learning and/or memory in the water maze test as hypothesized; this may have resulted from not including rats perinatally exposed to a lower dose of oxycodone in this experiment due to the small sample size of this group. Previous studies using prenatal morphine exposure models report conflicting results; memory and learning in rodents are either impaired or enhanced. Namely, juvenile rats prenatally exposed to morphine had impaired spatial memory in the Morris water maze or Y-maze test (38, 72, 73), but aberrant memories such as morphine reward memory in the conditioned place preference or forced swim tests were enhanced (49, 74–76). In contrast to our study, Davis et al. (39) found that prenatal exposure to oxycodone impaired spatial learning and memory in a battery of spatial tasks; in the Morris water maze, rats prenatally exposed to OXY had increased latency and greater distance traveled to find the platform when the intertrial interval was long, not short. Rats prenatally exposed to oxycodone also had a decreased use of spatial strategies and more use of non-spatial strategies such as wall-hugging. In addition, the retention of learning memory in the T-maze, assessed 5 days after acquisition of the training, was impaired. This finding is actually consistent with our report that although POE male rats were able to discriminate between the stress and non-stress cues during a classical conditioning paradigm on PD 40, they had impaired discrimination ability when retested on PD 75 (77). Moreover, rats prenatally exposed to oxycodone have more reference memory errors in the radial arm maze (39). These differences in results could be due to many reasons. In their study, the dose and route of administration of OXY were different than those used in the current experiment with escalating doses of OXY (10 mg/kg/day up to 15 mg/kg/day) *via* gavage for 28 days prior to breeding. In addition, the pups were reared by their biological mothers in their study, while surrogate fostering was used in the current study. A number of previous studies have shown that opiate administration negatively alters maternal rearing behavior toward the pups including measures such as cleaning of the pups, delay of maternal behaviors, and maternal aversion to pup odor (78, 79, 80). Neonatal rearing condition and neonatal maternal interaction such as maternal separation had long-term effects on the stress response of the offspring including an increase in restraint stress-induced norepinephrine release in the PVN in adult rats (81) and changes in the HPA axis at multiple levels that could be linked to epigenetic modification (82). Variations in maternal care also influence learning and behaviors of the offspring (83, 84). The adverse effects of neonatal maternal separation on the HPA axis were lessened by fostering the litters (85). Thus, fostering the pups when the dams were exposed to opiates could alter the HPA axis and possibly the neurobehavioral outcomes of the offspring. In addition, even though signs of withdrawal in the dams were monitored in the study by Davis et al., body weights of the OXY pups were approximately 10% lower than those of the controls, indicating possible neonatal opiate withdrawal and poorer nutritional status that can also affect long-term outcomes. In contrast, the body weights of the pups in our study were comparable in all groups.

In humans, prenatal exposure to opiates results in impairments in cognitive function and learning. Bunikowski et al. also reported that when evaluated at 1 year of age, children prenatally exposed to opiates had a mild psychomotor developmental impairment compared to the control group; these included impairments in “hearing and speech” and “intellectual performance” subscales (36). Guo et al. found that *in utero* opiate exposure was associated with impairments in the Auditory Rare Event Monitoring task and the Sternberg Memory task in children 7–12 years of age (35). More recently, Hunt et al. reported from their case–control study that infants prenatally exposed to opiates are more likely to experience neurodevelopmental impairments compared to healthy control infants, when assessed at 18 months and 3 years of age (37). The deleterious effects of prenatal opiate exposure on cognitive function persisted and did not decrease over time after controlling for permanent home placement and heroin use in the mother when children prenatally exposed to opiates were retested on the Wechsler Intelligence Scale for Children from 1 year old up to 8.5 years of age (86). These data suggest that prenatal exposure to opiates is associated with impaired cognitive functions. Therefore, although we did not detect any effects of POE on cognitive function, learning, and memory using a water maze as a paradigm, further investigation is warranted with different testing paradigms, testing at different ages, and with a lower dose of oxycodone.

Interestingly, we found that exposure to the lower dose of oxycodone of 0.5 mg/kg/day but not the higher dose of 2.0 mg/kg/day was associated with hyperactivity in the offspring. This difference could be due to the development of tolerance to the higher dose of oxycodone. Opiate tolerance is characterized to be pharmacodynamic, time and dose-dependent, and opioid receptor specific (87). Tolerance can develop after exposure to a KOR agonist (88) even for as short as 5 days (17). Whether or not tolerance to the stimulatory effects on the HPA axis by KOR agonists is dose-dependent has not been well studied, but tolerance to MOR agonists was dose-related (89). It is possible that in our study, rat dams that were exposed to the higher dose of oxycodone (2 mg/kg/day) may have developed opioid tolerance leading to a decreased fetal CORT exposure, thus there were fewer effects on the developing HPA axis and the neurodevelopmental outcomes of the offspring.

In summary, POE was associated with hyperactivity in young adult rats. In these studies, perinatal oxycodone treatment had no effect on the responses of pups isolated from their dams, or in learning and memory deficits, which could be due to the small sample sizes, testing paradigms, or the doses of oxycodone tested. These issues remain to be resolved.

## Ethics Statement

This study was carried out in accordance with the recommendations of the University of Kentucky Institutional Animal Care and Use Committee. The protocol was approved by the University of Kentucky Institutional Animal Care and Use Committee.

## Author Contributions

TS: designed and conducted the studies, analyzed the data, wrote the manuscript, PI of the funding. SL and MW: assisted in designing, conducting, and analyzing the data and edited the manuscript. PW: statistician, responsible for all data analysis. KW: conducted and analyzed the data. HB: assisted in designing and analyzing the data. SB: main assistant in designing, conducting, and analyzing the data and edited the manuscript.

## Conflict of Interest Statement

The authors declare that the research was conducted in the absence of any commercial or financial relationships that could be construed as a potential conflict of interest.
